# The Effect of immunonutrition in patients undergoing pancreaticoduodenectomy: a systematic review and meta-analysis

**DOI:** 10.1186/s12885-023-10820-7

**Published:** 2023-04-17

**Authors:** Yinyin Fan, Nianxing Li, Jing Zhang, Qiaomei Fu, Yudong Qiu, Yan Chen

**Affiliations:** 1 Department of Biliary-Pancreatic Surgery, Nanjing Drum Tower Hospital Clinical College of Jiangsu University, Nanjing, 210008 China; 2 Department of Biliary-Pancreatic Surgery, Nanjing Drum Tower Hospital, Affiliated Hospital of Medical School, Nanjing University, Nanjing, 210002 China; 3 Department of Nursing, Nanjing Drum Tower Hospital, Affiliated Hospital of Medical School, Nanjing University, Nanjing, China

**Keywords:** Pancreaticoduodenectomy, Immunonutrition, Prognosis, Length of hospital stay, Infectious complications

## Abstract

**Background:**

Pancreaticoduodenectomy (PD) is a complex and traumatic abdominal surgery with a high risk of postoperative complications. Nutritional support, including immunonutrition (IMN) with added glutamine, arginine, and ω-3 polyunsaturated fatty acids, can improve patients’ prognosis by regulating postoperative inflammatory response. However, the effects of IMN on PD patients’ outcomes require further investigation.

**Methods:**

PMC, EMbase, web of science databases were used to search literatures related to IMN and PD. Data such as length of hospital stay, infectious complications, non-infectious complications, postoperative pancreatic fistula (POPF), delayed gastric emptying (DGE), mortality, systemic inflammatory response syndrome (SIRS) duration, IL-6, and C-reactive protein (CRP) were extracted, and meta-analyses were performed on these data to study their pooled results, heterogeneity, and publication bias.

**Results:**

This meta-analysis involved 10 studies and a total of 572 patients. The results showed that the use of IMN significantly reduced the length of hospital stay for PD patients (MD = -2.31; 95% CI = -4.43, -0.18; P = 0.03) with low heterogeneity. Additionally, the incidence of infectious complications was significantly reduced (MD = 0.42; 95% CI = 0.18, 1.00, P = 0.05), with low heterogeneity after excluding one study. However, there was no significant impact on non-infectious complications, the incidence of POPF and DGE, mortality rates, duration of SIRS, levels of IL-6 and CRP.

**Conclusion:**

The use of IMN has been shown to significantly shorten hospital stays and decrease the frequency of infectious complications in PD patients. Early implementation of IMN is recommended for those undergoing PD. However, further research is needed to fully assess the impact of IMN on PD patients through larger and higher-quality studies.

**Supplementary Information:**

The online version contains supplementary material available at 10.1186/s12885-023-10820-7.

## Background

Pancreatoduodenectomy (PD) is a classic surgical approach to treat tumors of the head of the pancreas, ampulla, and distal bile ducts. It has been widely described as a major traumatic operation in abdominal surgery, resulting in high post-operative mortality. PD involves the resection of multiple organs and the reconstruction of the gastrointestinal tract, pancreas-intestine, biliary-intestinal and other gastrointestinal tracts, and the postoperative morbidity rates remain high [1]. Several feasible clinical scores and biomarkers have been proposed aiming at timely predicting the risk of developing severe complications, such as clinically relevant pancreatic fistula, and optimally managing in-hospital patients [2–4]. Patients with metabolic, nutritional, or immunodeficiency disorders may be at heightened risk of complications during or after PD, potentially affecting their ability to achieve full recovery [5, 6].

Nutritional support, as an adjuvant therapy in the routine perioperative period, improves the prognosis of patients and prolongs their lifespan. Currently, nutritional support has been recommended as the first-line treatment [7]. Immunonutrition (IMN) is a type of nutritional support that utilizes specific nutrients to control postoperative inflammatory responses and counteract postoperative immune dysfunction. Commonly used IMN include glutamine, arginine, omega-3 polyunsaturated fatty acids, and nucleotides [8, 9]. For patients undergoing PD surgery, the current global implementation of the Enhanced Recovery After Surgery (ERAS) protocol recommends the use of preoperative or perioperative medical nutritional regimens, which may include IMN comprising of arginine, omega-3 fatty acids, and nucleotides, administered in the period of 5 to 7 days prior to the surgery [10, 11].

Several studies have introduced the impact of IMN between patients underwent in gastrointestinal surgery [12, 13], but the role of IMN in postoperative outcomes after PD remains unclear. Therefore, this systematic review and meta-analysis of the current literature aims to evaluate the use of IMN support in PD patients, gaining a more comprehensive understanding of the role of IMN in patients receiving PD.

## Methods

### Search strategy

This systematic review and meta-analysis was conducted in accordance with the Preferred Reporting Items for Systematic Reviews and Meta-Analyses (PRISMA) statement. Published literature was systematically searched using PMC, Embase, and Web of Science up to 31 August 2022). The keywords (“Nutrition Therapy” or “Nutritional Support” or " immunonutrition “) and (“Pancreatic Neoplasms” or “pancreatic cancer “or” Pancreatoduodenectomy “or “Pancreatic surgery”)) were used to search the above databases. The retrieved relevant literature information was imported into the literature management software Note Express, and two researchers (YYF and NXL) independently screened all relevant articles to identify those that met the inclusion criteria. In the event of a disagreement, a third researcher (JZ) made the final decision on the inclusion of the article.

### Inclusion and exclusion criteria

Literatures included in this meta-analysis needed to meet the following criteria: (1) included patients who underwent pancreatoduodenectomy (2) studies comparing IMN (including oral, enteral, and parenteral nutrition) with standard nutrition supplementation (conventional nutritional supplements) differences. (3) The IMN group used at least one IMN component (arginine, glutamine, omega-3 fatty acids, and/or nucleotides); (4) a control group that did not receive any IMN was included in the study. (5) The results of the study included at least one prognostic outcome, such as postoperative complications, mortality, and length of hospital stay. The exclusion criteria are as follows: (1) The types of literature are review, commentary, conference abstract, and case report. (2) The research is not based on clinical trials on patients but on animal experiments or in vitro experiments. (3) No corresponding prognostic indicators were provided. (4) Immunonutrition as postoperative enteral nutrition. (5) The article was not published in English.

### Data extraction

The data information of each study was extracted, including: first author’s name, publication year, country, study type, number of patients and controls, type of surgery, immune nutrition composition, time of using immune nutrition, prognosis, length of hospital stay, complications, Postoperative systemic inflammatory response syndrome (SIRS) duration, IL-6 and CRP. Data extraction was performed independently by two researchers (YYF and NXL). In case of any disagreements, a consensus was reached through discussion.

### Risk of bias assessment of included studies

The risk of bias of each included study was assessed using the updated Risk of Bias tool (RoB-2 tool) provided by the Cochrane Collaboration, which included an assessment of five items (Randomization process, deviations from intended interventions, missing outcome data, measurement of the outcome, selection of the reported result), data assessment was carried out independently by two researchers (YYF and NXL), and in the event of any discrepancies, a consensus was reached through discussion. Finally, high risk, low risk, or some concerns are assessed. The assessment involves identifying potential biases or limitations in study design, conduct, analysis, and reporting that may affect the validity of the study results.

### Statistical analysis

RevMan 5.3 software was used for statistical analysis and graphing. The combined effect size and pooled effect size of each included study were displayed using a forest plot. The heterogeneity of each study was assessed according to I^2^, with I^2^ < 25% indicating low heterogeneity, I^2^ between 25 and 50% indicating moderate heterogeneity, and I^2^ > 50% indicating significant heterogeneity. Different effect models were used depending on I^2^ and P values. When I^2^ > 50%, a random effects model is used. When I² < 50%, a fixed effects model was used. Sensitivity analyses were conducted to assess the stability of the results by excluding one study at a time. When the number of included studies exceeded 5, potential publication bias was assessed using funnel plots.

## Results

### Characteristics of selected studies

**Fig. 1:**
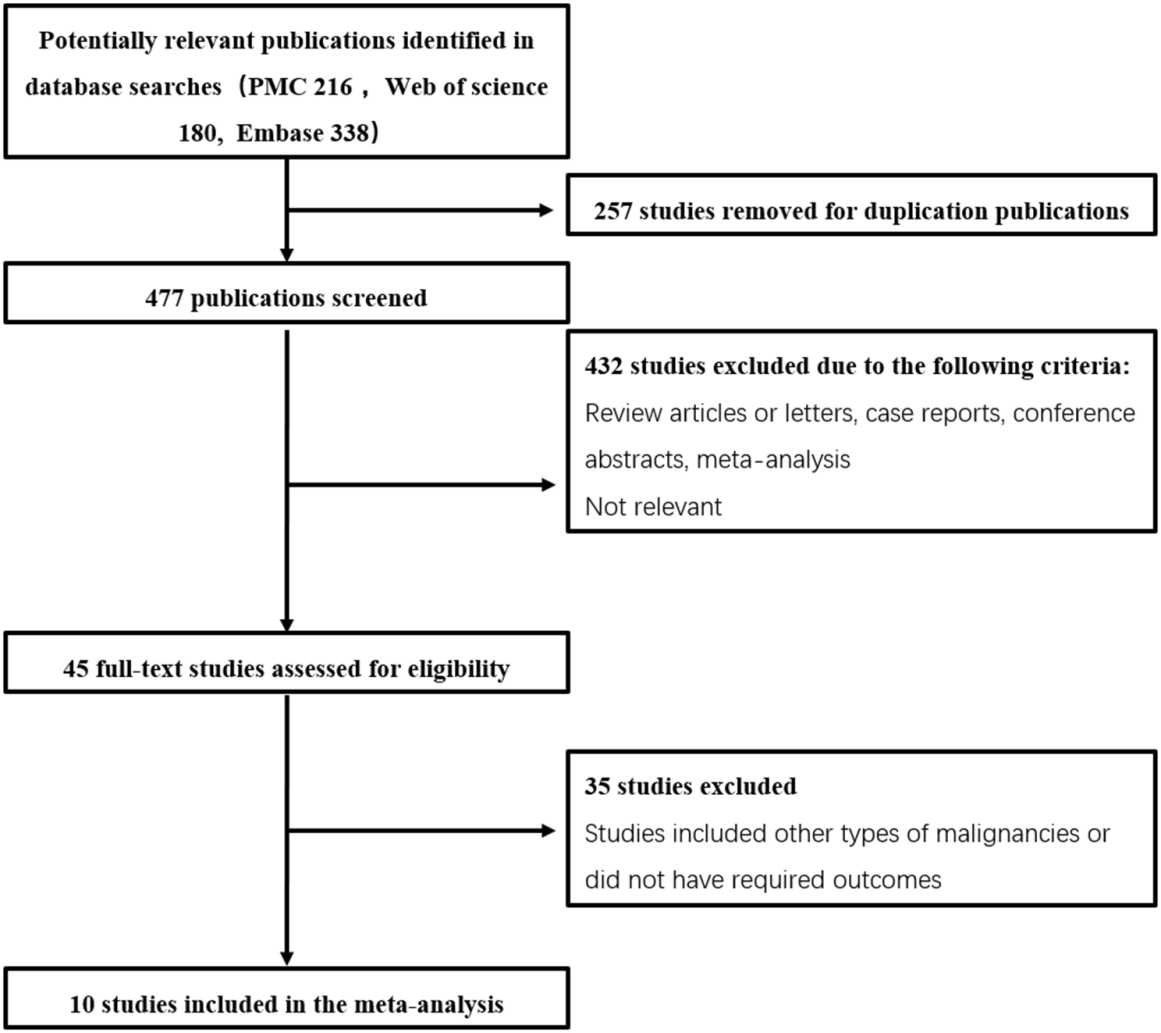
The flow chart of the selection process

In this study, a total of 734 articles were retrieved by searching PMC, Web of science and Embase. After removing 257 duplicate studies, the titles and abstracts of 477 studies were screened. We conducted a further full-text evaluation of 45 studies, of which 35 studies were excluded because they included other types of tumors or did not provide relevant prognostic data. Figure 1 presents a flowchart illustrating the literature search and screening process, including the number of articles retrieved, screened, and excluded at each stage, leading to the final selection of 10 studies for the meta-analysis [14–23].

**Fig. 2:**
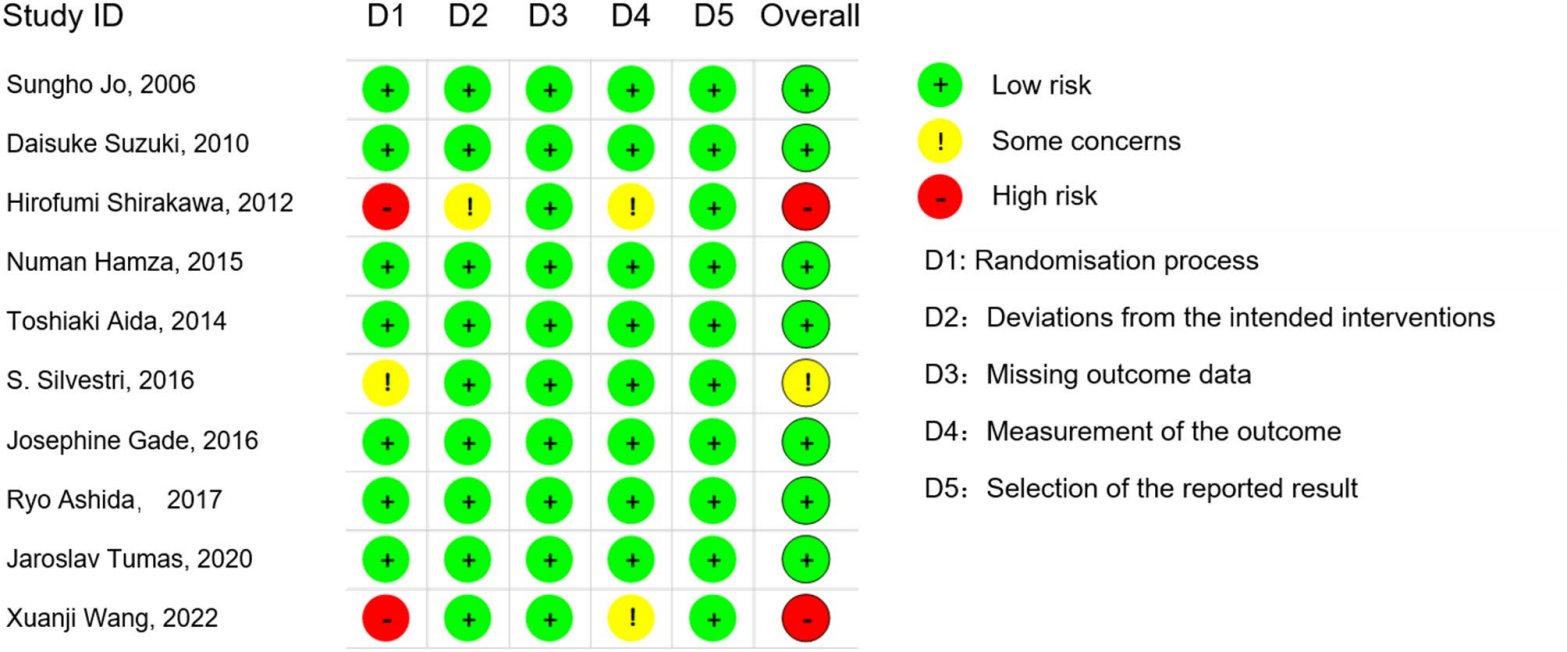
Risk of bias assessment of included studies

This meta-analysis comprised 10 studies, consisting of 9 randomized controlled trials (RCTs) and one retrospective study (*Xuanji Wang, et al.*, 2022), conducted between 2006 and 2022, with a total of 572 participants. Of these participants, 293 were PD patients who received IMN, while 279 were control PD patients who did not receive IMN. The studies originated from Europe (4), Asia (5), and the Americas (1). IMN in the meta-analysis included Glutamine, Arginine, omega-3 fatty acids, and RNA. The two administration methods were oral and parenteral nutrition. All ten studies included in the analysis administered IMN prior to surgery. Table 1 provides a comprehensive summary of the key characteristics and outcomes of the studies included in the analysis. The risk of bias of the included studies was assessed using the RoB-2 tool. Two studies were assessed as having a high risk of bias due to inadequate measurement of the outcome and lack of randomization. One study was assessed as having some concerns due to inadequate measurement of the outcome or lack of randomization. The remaining seven studies were assessed as having a low risk of bias (Fig. 2).

**Table 1:** Characteristics of the Included studies

Study	Characteristics of enrolled patients				Immune Nutrition Components	Method of Administration	Outcomes	Main results
	No. (patients/controls)	Age	BMI	Tumor type and stage (No.)				
Sungho Jo, 2006	32/28	56.8 ± 9.4 ^1^	NA	Tumors of the pancreas, bile duct, ampulla, and duodenum	Glutamine	Parenteral nutrition. The Gln group received 10ml/kg/day of Glamin (containing 0.2 g Gln) for 7 days, starting from 2nd day before to 5th day after the surgery.	LOS, mortality, CRP, POPF, DGE	No significant beneficial effect of Gln supplementation with a low-dose parenteral regimen was demonstrated on the surgical outcome after a PD for periampullary tumors.
Daisuke Suzuki, 2010	10/10	62 ± 4 ^2^	NA	Tumors of the pancreas, bile duct and ampulla, TNM I/II (4/4)	Arginine, ω–3 fatty acids, and RNA	Oral supplementation. A perioperative group, oral supplementation for 5 days (1,000 kcal/day) before operative resection with a formula enriched with arginine, omega-3 fatty acids, and RNA.	Infectious complications, noninfectious complications, mortality, SIRS, IL-6, POPF	In the perioperative group, the rate of infectious complications was significantly reduced compared with that in the other groups.
Hirofumi Shirakawa, 2012	18/13	62.6 ± 8.5 ^1^	21.9 ± 2.1 ^1^	No. of PIDC/BDC/others (4/5/9)	Arginine, ω–3 fatty acids, and RNA	Oral supplementation. The patients were instructed to consume 3 packs/day (750 mL, 9.6 g of arginine, 2.49 g of omega-3 fatty acids, and 0.96 g of RNA) of Impact Japanese version in addition to their normal diets over a 5-day period immediately before surgery.	LOS, mortality, CRP, SIRS, POPF, DGE	Preoperative ingestion of Impact appeared effective in preventing wound infections and reducing surgical stress responses.
Numan Hamza, 2014	17/20	63(58–69) ^3^	27.0 (25.3–28.7) ^3^	Pancreatic/Ampullary cancer/Duodenal cancer/Ductal atypia (10/4/1/2)	Arginine, ω–3 fatty acids, and RNA	Oral supplementation. Patients were asked to consume 3 cartons (200 mL per carton) of either feed per day for 14 days before surgery (IMPACT feed contains arginine 1.9 g/100 mL, mRNA 0.255 g/100 mL, Omega-3-fatty acids 0.5 g/100 mL).	IL-6	Administering EIMN rather than SEN perioperatively is associated with a favorable modulation of the inflammatory response.
Toshiaki Aida, 2014	25/25	66.4 ± 1.5 ^2^	21.5 ± 0.5 ^2^	Tumors of pancreatic/bile duct/ampullary/0thers (14/4/3/4), TNM 0/I/II/III/IV (2/3/10/5/1)	Arginine, ω–3 fatty acids, and RNA	Oral supplementation. Patients in the IN group received oral supplementation (1,000 kcal/day) containing arginine, u-3 fatty acids, and RNA for 5 days before surgery.	Infectious complications, noninfectious complications, mortality, IL-6, SIRS, POPF, DGE	The IMN group had a lower infectious complication rate and less severe complications compared to the control group.
S. Silvestri, 2016	48/48	62.27 ± 11.45 ^4^	24.5 ± 3.5 ^4^	Tumors of pancreatic/ampullary/biliary/others(28/6/8/6)	Arginine, ω–3 fatty acids, and RNA	Oral supplementation. Preoperative IN supplemental liquid diet (Oral Impact, L-arginine 1.8 g, RNA 0.2 g, ommege-3 fatty acids 0.6 g) for at least 5 days before pancreatic surgery.	LOS, infectious complications, mortality, POPF, DGE	Preoperative oral IMN reduces infection risk and hospital stay duration for well-nourished PD patients.
Josephine Gade, 2016	19/16	68 (50–81) ^5^	24.3 (18.8–28.3) ^5^	Pancreatic cancer/benign pancreatic tumor (24/11)	Arginine, ω–3 fatty acids, and RNA	Oral supplementation. The intervention group received 7 days of preoperative oral IN, Oral Impact Powder as a supplement to their normal diet to reach a total goal of 1.5 g protein/kg.	LOS, infectious complications, mortality	Adding IMN to the diet preoperatively with the goal of achieving 1.5 g protein/kg body weight did not result in significant clinical benefits for patients scheduled for pancreatic surgery
Ryo Ashida, 2019	11/9	64 ± 11 ^2^	55.9 ± 13.5 ^2^	Tumors of the pancreas/bile duct/ampulla/others (3/2/3/3)	ω–3 fatty acids	Oral supplementation. Patients in the treatment group received oral supplementation (600 kcal/day) containing EPA for 7 days before surgery, in addition to 1,200 kcal of regular food.	Infectious complications, IL-6, POPF	Preoperative IMN had limited effect on the occurrence of postoperative hypercytokinemia or infectious complications in patients undergoing PD
Jaroslav Tumas, 2020	30/40	62.6 ± 10.5 ^1^	26.8 ± 5.6 ^1^	PDAC/others (17/13)	L-arginine and polyunsaturated fats	Oral supplementation. IN group received 5 days of preoperative IN (L-arginine 6.04 g/day and polyunsaturated fat 4 g/day) in addition to the usual preoperative nutritional management.	IL-6, CRP	IMN may be more beneficial for patients with PDAC than those with benign pancreatic diseases or less aggressive tumors, regardless of their nutritional status.
Xuanji Wang, 2022	83/70	66 ^6^	26.1 ^6^	PDAC/others (83/125)	Arginine, ω–3 fatty acids, and RNA	Oral supplementation. Patients were given and instructed to take IMPACT for 5 days, 3 times daily, prior to surgery.	Infectious complications, POPF, DGE	Preoperative IMN had no effect on LOS or infections in PDAC patients undergoing PD. However, in non-PDAC patients, it was linked to longer LOS and higher rates of intraabdominal infections

Abbreviations: IMN, Immunonutrition. RCT, Randomized controlled trial. PD, Pancreaticoduodenectomy. ω–3, Omega-3. LOS, Length of hospital stay. SIRS, Systemic inflammatory response syndrome. CRP: C-reactive protein. PIDC, Pancreatic invasive ductal carcinoma. BDC, Bile duct carcinoma. EPA, Enriched eicosapentaenoic acid. PDAC, Pancreatic ductal adenocarcinoma. POPF, Postoperative pancreatic fistula. DGE, Delayed gastric emptying. ^1^ Mean ± SD. ^2^ Mean ± SEM. ^3^ Mean (95% confidence interval). ^4^ Median ± SD. ^5^ Median (range). ^6^ Mean

### The impact of IMN on the length of hospital stay

**Fig. 3:**
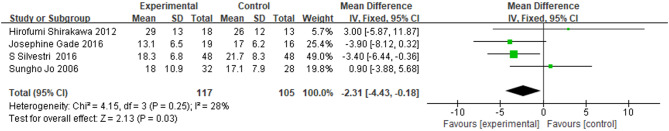
**Meta-analysis of the effect of IMN on hospitalization days** Forest plot of length of hospital stay. The random-effects model was used. The square size of individual studies represented the weight of the study. Vertical lines represent 95% CI of the pooled estimate. The diamond represents the overall summary estimate, with the 95% CI given by its width

Out of the 10 studies, four studies reported data on the length of hospital stay, with a total of 117 patients receiving IMN and 105 control patients who did not receive IMN. Pooled data from forest plots showed that the IMN group had significantly less hospital stay than the control group (MD = -2.31; 95% CI = -4.43, -0.18; P = 0.03), with moderate heterogeneity across studies (I^2^ = 28%, P = 0.25) (Fig. 3).

### The impact of IMN on postoperative infectious complications

**Fig. 4:**
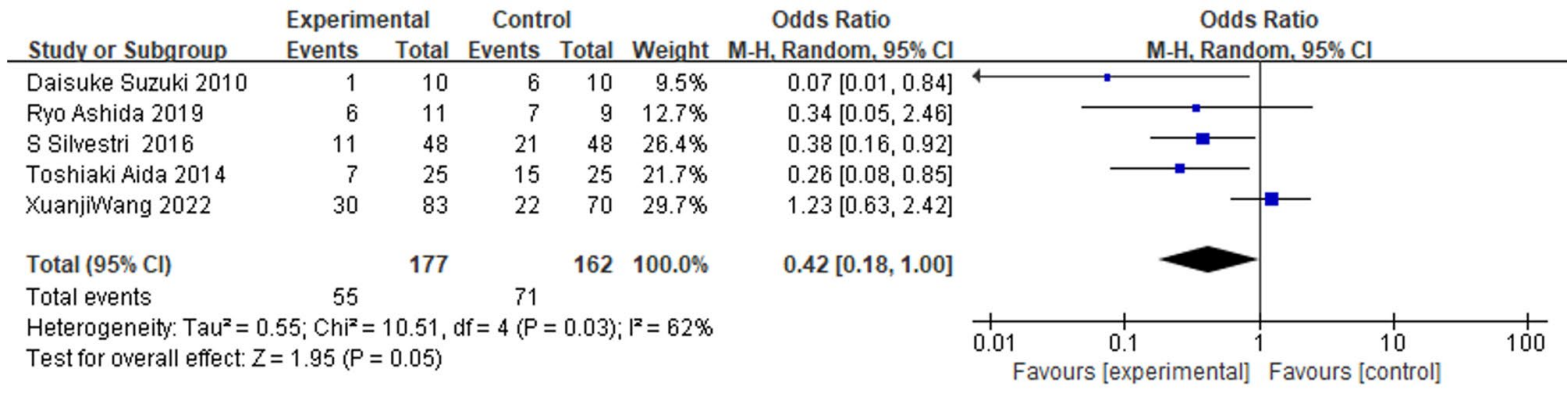
**Meta-analysis of the effect of IMN on the incidence of infectious complications** Forest plot of infectious complications. The random-effects model was used. The square size of individual studies represented the weight of the study. Vertical lines represent 95% CI of the pooled estimate. The diamond represents the overall summary estimate, with the 95% CI given by its width

The incidence of infectious complications was assessed in five studies comparing the IMN and control groups. A total of 339 patients were included in the meta-analysis, of which 177 received IMN and 162 were in the control group. The pooled analysis of forest plots showed a significant reduction in the incidence of infectious complications in the IMN group compared to the control group (OR = 0.42; 95% CI = 0.18, 1.00, P = 0.05), with significant heterogeneity (I^2^ = 62%, P = 0.03), and a random effects model was used (Fig. 4). Sensitivity analysis indicated that the study by *Xuanji Wang, et al.* was the source of heterogeneity among studies. After its removal, the pooled effect was (OR = 0.30; 95% CI = 0.16, 0.57; P = 0.0002), with no significant heterogeneity (I^2^ = 0%, P = 0.65).

### The impact of IMN on postoperative non-infectious complications

**Fig. 5:**
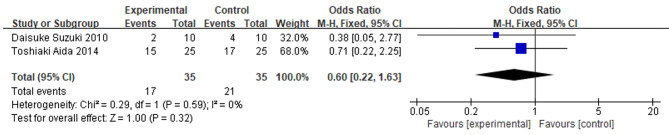
**Meta-analysis of the effect of IMN on the incidence of non-infectious complications** The forest plot displays the incidence of non-infectious complications, with the fixed-effects model used for the analysis. The plot provides a visual representation of the effect sizes and confidence intervals for each study included in the analysis, allowing for a comparison of the results and assessment of the overall effect of IMN on non-infectious complications following surgery

Two studies reported data on the incidence of noninfectious complications in both the IMN and control groups. A total of 35 patients receiving IMN and 35 control patients were included in these studies. A pooled analysis of forest plots showed no significant difference in the incidence of infectious complications between the IMN group and the control group (OR = 0.60; 95% CI = 0.22, 1.63; P = 0.32). There was no heterogeneity among the studies (I^2^ = 0%, P = 0.59) (Fig. 5).

### The impact of IMN on the incidence of postoperative pancreatic fistula (POPF) and delayed gastric emptying (DGE)

**Fig. 6:**
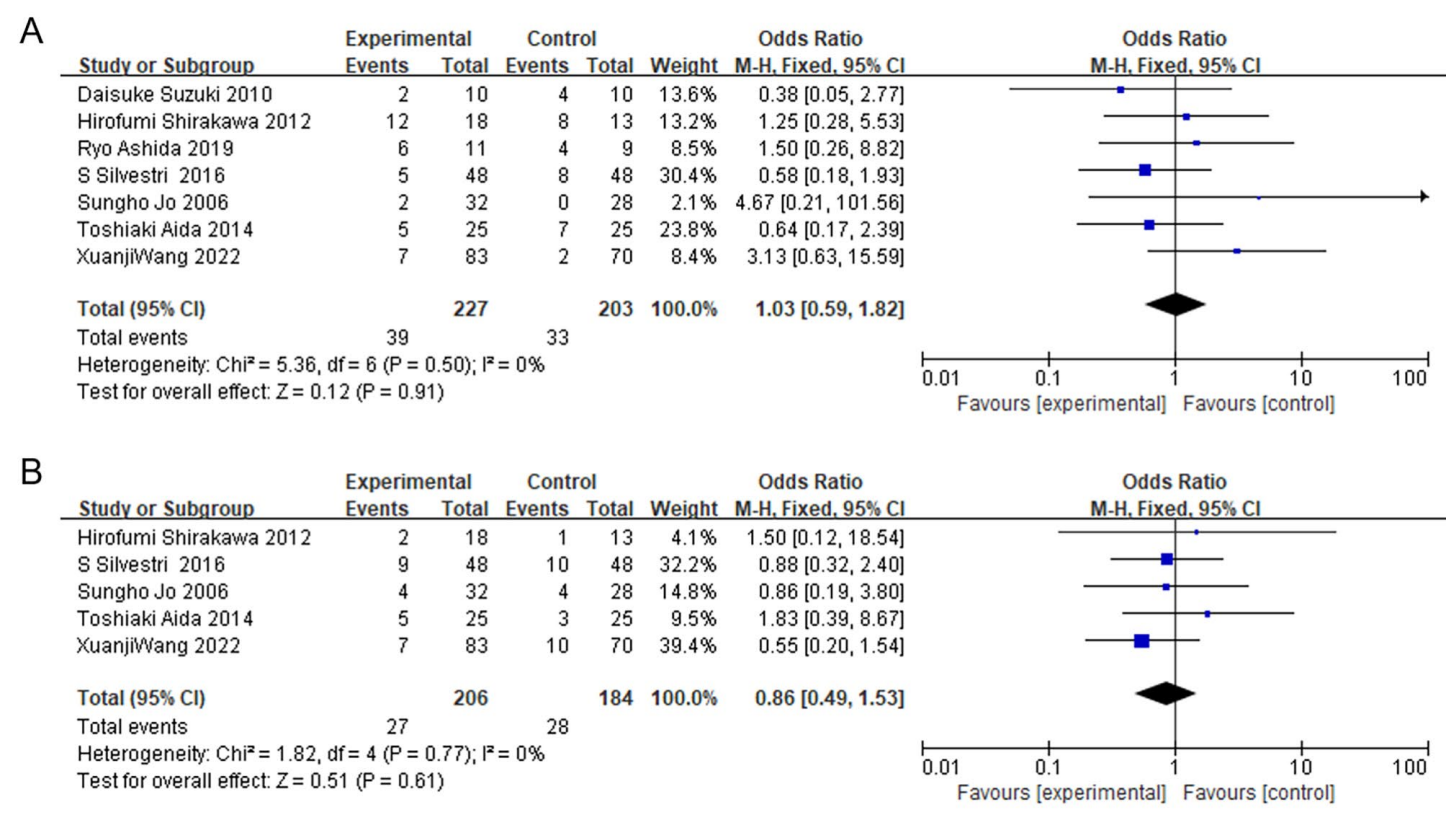
**Meta-analysis of the effect of IMN on the incidence of POPF and DGE** The forest plot shows the incidence of postoperative pancreatic fistula (POPF) and delayed gastric emptying (DGE) as separate panels (A and B), with the fixed-effects model employed to analyze the data

Seven studies were included in the meta-analysis to assess the effect of IMN on POPF. The pooled data from the forest plot indicated that there was no significant difference in the incidence of POPF between the IMN group and the control group (OR = 1.03; 95% CI = 0.59, 1.82; P = 0.91), and the studies were homogeneous (I^2^ = 0%, P = 0.50) (Fig. 6A). Similarly, the meta-analysis of 5 studies showed that there was no statistically significant difference in the incidence of DGE between the IMN group and the control group (OR = 0.86; 95% CI = 0.49, 1.53; P = 0.61), and there was no significant heterogeneity among the included studies (I^2^ = 0%, P = 0.77) (Fig. 6B). The funnel plot exhibited a symmetrical distribution of studies, indicating the absence of significant publication bias (Figure S1).

### The impact of IMN on postoperative mortality

**Fig. 7:**
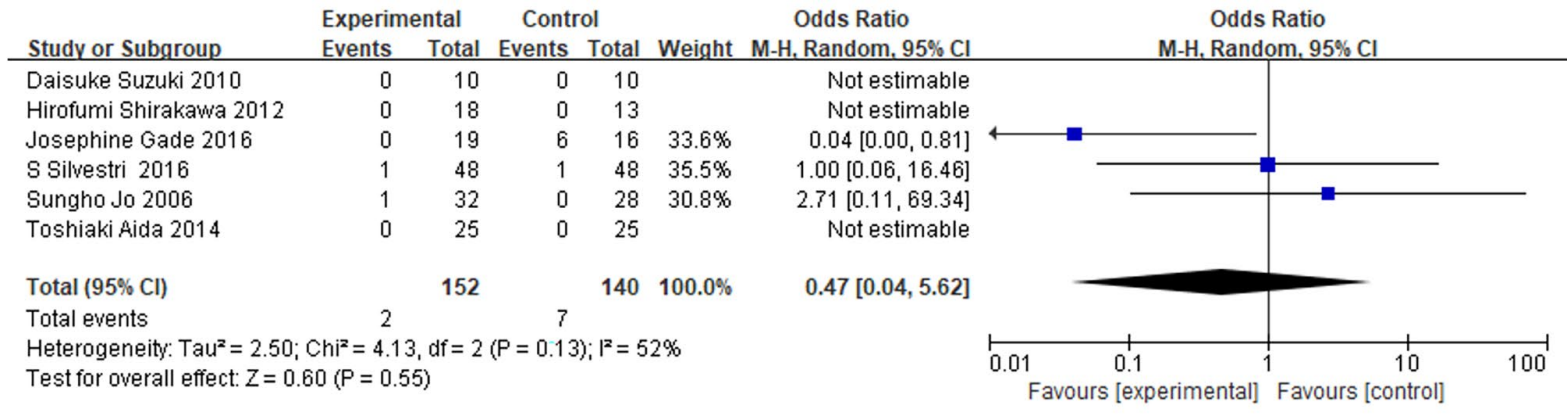
**Meta-analysis of the effect of IMN on postoperative mortality** Forest plot of postoperative mortality, with the random-effects model used for the analysis. The random-effects model accounts for the potential heterogeneity among the studies and provides a more conservative estimate of the overall effect size

Mortality was evaluated in six studies, while death was reported in three studies. The total sample size included 152 patients receiving IMN and 140 control patients across these studies. The pooled analysis of forest plots indicated no significant difference in mortality between the IMN group and the control group (OR = 0.47; 95% CI = 0.04, 5.62; P = 0.55), and due to mild heterogeneity between studies (I^2^ = 52%, P = 0.13), the random effects model was used for the pooled data (Fig. 7).

### The impact of IMN on the duration of postoperative SIRS

**Fig. 8:**
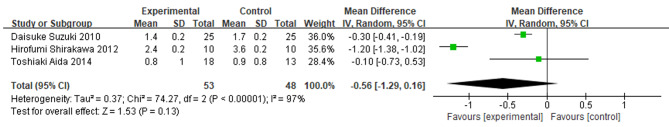
**Meta-analysis of IMN on postoperative SIRS duration** Forest plot of postoperative SIRS duration, with the random-effects model used for the analysis

The analysis included data on the duration of SIRS from three studies comprising 53 patients receiving IMN and 48 control patients. The pooled forest plot analysis revealed that there was no statistically significant difference in the duration of SIRS between the IMN group and the control group (MD = -0.56; 95% CI = -1.29, 0.16; P = 0.13). However, there was significant heterogeneity observed across studies (I^2^ = 97%, P < 0.00001), thus the random effects model was employed to pool the data. (Fig. 8).

### The impact of IMN on postoperative immune-related indicators

**Fig. 9:**
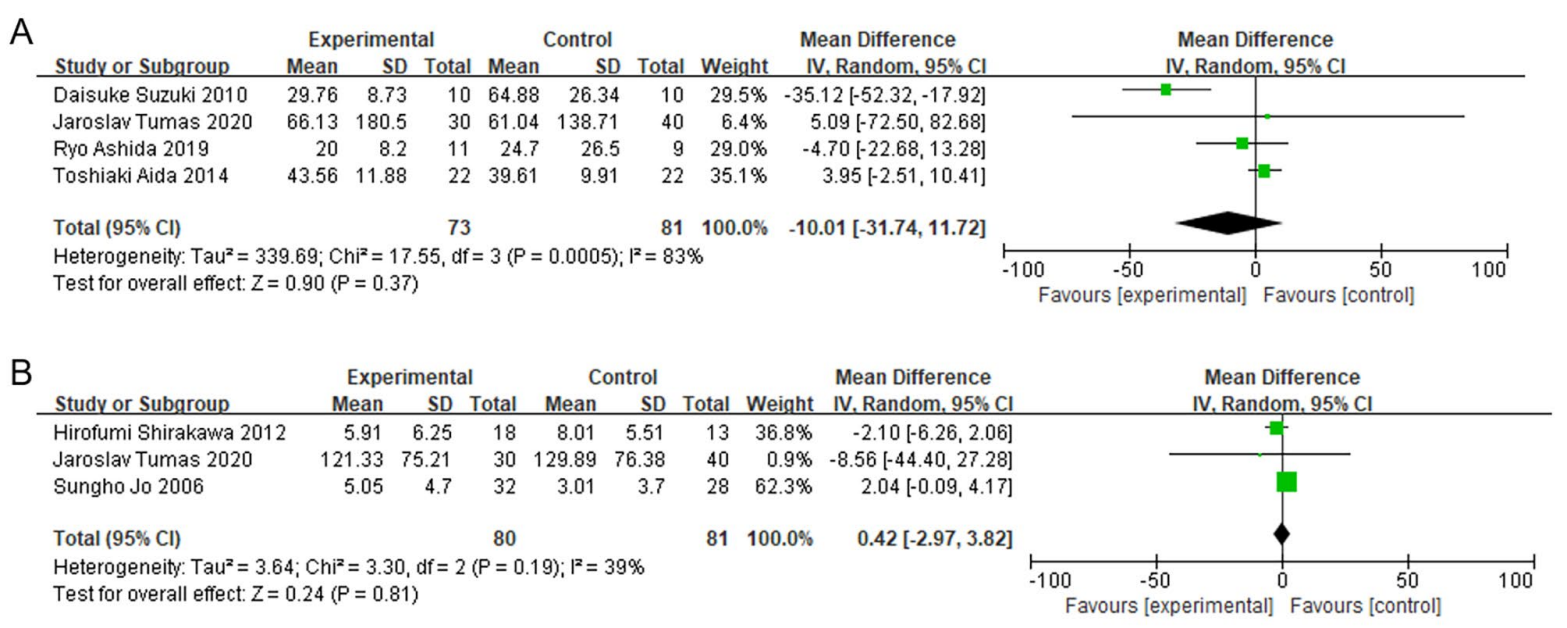
**Meta-analysis of the effect of IMN on IL-6 and CRP** Forest plot depicting the levels of IL-6 and CRP analyzed separately (A and B) using a random-effects model

We also investigated the impact of IMN on immune-related markers IL-6 and CRP. The pooled data did not reveal significant difference in IL-6 levels between the IMN and control groups (MD = -10.01; 95% CI = -31.74, 11.72; P = 0.37), and high heterogeneity was observed among the studies (I^2^ = 83%, P = 0.0005) (Fig. 9A). Similarly, there was no significant difference in CRP levels between the IMN and control groups, as indicated by the pooled data (MD = 0.42; 95% CI = -2.97, 3.82; P = 0.81), and moderate heterogeneity was observed among the included studies (I^2^ = 39%, P = 0.19) (Fig. 9B).

## Discussion

This systematic review and meta-analysis aimed to investigate the effects of IMN on various outcomes, including postoperative hospital stay, infectious and non-infectious complications, POPF, DGE, SIRS duration, mortality, and immune and inflammatory markers. Our findings suggest that IMN is effective in reducing hospital stay duration and postoperative infectious complications among PD patients. However, we did not observe a significant effect on non-infectious complications, POPF, DGE, SIRS duration, mortality, or immune and inflammatory markers such as IL-6 and CRP.

Pancreatic cancer patients commonly experience nutritional abnormalities and cachexia, with up to 85% reporting malnutrition and nearly 71% of those with pancreatic and periampullary cancers experiencing cachexia, which is associated with a higher mortality rate [24, 25]. Thus, the nutritional status of patients undergoing pancreaticoduodenectomy should be closely monitored. Additionally, patients with these malignancies often exhibit immune dysfunction, which may contribute to higher rates of postoperative complications and mortality. IMN is a type of nutritional support that involves the use of specific nutrients, such as arginine, glutamine, omega-3 polyunsaturated fatty acids and nucleotides. Arginine is a semi-essential amino acid for catabolism and plays an important role in protein synthesis. Arginine can promote the proliferation and activity of T cells and stimulate the phagocytosis of neutrophils [26]. Furthermore, arginine may help to reduce inflammation by inhibiting the production of cytokines such as TNF-alpha and IL-6 [27]. Glutamine is a conditionally essential amino acid that can become depleted during periods of stress or infection. It has been shown to regulate immune functions such as lymphocyte proliferation, cytokine production, and may help reduce mucosal damage during cancer treatment [28, 29]. Omega-3 fatty acids, namely eicosapentaenoic acid (EPA) and docosahexaenoic acid (DHA) [30], omega-3 fatty acids can significantly reduce the production of pro-inflammatory cytokines, reduce the expression of cell adhesion molecules on lymphocytes and monocytes, and promote the resolution of inflammation [31, 32]. Exogenous nucleotides have been shown to support immune function by promoting the maturation, activation and proliferation of lymphocytes, increasing antibody production, and enhancing cellular immunity [33]. Patients who undergo PD often experience poor nutritional status and immune dysfunction, which can increase the risk of postoperative complications. Therefore, the use of IMN after surgery may theoretically benefit these patients by providing key nutrients to support immune function and promote recovery.

Although several studies have investigated the effects of IMN in PD patients, the results have been inconsistent and difficult to interpret due to variations in study design and patient populations [17, 34, 35]. Therefore, a comprehensive meta-analysis was necessary to synthesize the available evidence and provide more conclusive results. While a previous meta-analysis evaluated the impact of IMN in pancreatic cancer patients undergoing surgery, it only included six studies [36]. Our meta-analysis, which included a larger number of studies, found that IMN significantly shortened postoperative hospital stays and reduced the incidence of infectious complications, with low heterogeneity across studies. These findings are consistent with previous research [36, 37]. Pooled data from this study showed that IMN had little effect on non-infectious complications and mortality, which was also consistent with other studies [36, 37]. After undergoing PD, the body’s immune system can be challenged and compromised, increasing the risk of postoperative infections. IMN has been shown to help regulate immune function and reduce the incidence of infectious complications. However, non-infectious complications are typically associated with factors such as the surgery itself, anesthesia, and other factors, which are not directly related to immune function. Similar to certain studies after resection of gastrointestinal cancer [38, 39], IMN may significantly reduce the risk of infectious complications following PD. However, its impact on non-infectious complications, such as POPF and DGE, appears to be insignificant. SIRS is a series of cascaded inflammatory responses produced by the body to trauma [40]. However, the effect of IMN on SIRS duration is still unclear. In this meta-analysis, we found a non-significant trend towards decreased SIRS duration with IMN, but the heterogeneity was high, and only three studies provided data on this outcome. Thus, further studies with more data are needed to confirm the effect of IMN on SIRS duration.

IL-6 and CRP are crucial biomarkers closely linked to postoperative inflammation. The pooled data analysis did not show a significant effect of IMN on IL-6 and CRP levels, and there was heterogeneity among the studies, which may be due to differences in the composition and dose of the IMN formulations used, as well as variations in individual patient factors such as age and baseline nutritional status. The literature included limited data on IL-12 and TGF-β, which are also important inflammation-related cytokines. CD26 is a multifunctional cell surface glycoprotein that can be induced and upregulated by IL-12 [41]. CD26 can also exist in a soluble form in plasma, and its expression can be used as a diagnostic and prognostic marker for gastrointestinal tumors [42–44]. Therefore, investigating the effect of IMN on these inflammation-related cytokines would be a worthwhile research direction.

This study has several limitations. Despite including 10 studies, the prognostic measures assessed within each study were not consistent. Data on non-infectious complications, the duration of SIRS, and immune markers such as CRP, IL-6, CD4, and CD8 are lacking. Additionally, due to the small number of studies included, subgroup analyses of these data were not possible. Moreover, limited data on the dose and timing of IMN use in each study made it challenging to determine the optimal dosage and duration of IMN effects.

In summary, our meta-analysis investigated the effect of IMN on PD patients and found that it significantly reduces the length of hospital stay and the incidence of infectious complications. However, no significant effect was observed on non-infectious complications, POPF, DGE, mortality, CRP and IL-6. Our findings suggest that IMN may benefit PD patients, but large-scale, high-quality randomized controlled studies are still needed to more comprehensively evaluate its role.

## Electronic supplementary material

Below is the link to the electronic supplementary material.

Supplementary Material 1

## Data Availability

The datasets used and/or analysed during the current study available from the corresponding author on reasonable request.

## Abbreviations

IMN: Immunonutrition
PD: Pancreaticoduodenectomy
ERAS: Enhanced Recovery After Surgery
SIRS: Systemic inflammatory response syndrome
CRP: C-reactive protein
RCT: Randomized controlled trial
OR: Odds ratio
MD: Mean difference
PODF: Postoperative pancreatic fistula
DGE: Delayed gastric emptying
EPA: Eicosapentaenoic acid
DHA: Docosahexaenoic acid
