# Supplementary material for: The Effect of immunonutrition in patients undergoing pancreaticoduodenectomy: a systematic review and meta-analysis
Source: BMC Cancer. 2023 Apr 17;23:351. doi: 10.1186/s12885-023-10820-7 (PMC10108524; doi:10.1186/s12885-023-10820-7)
Supplement: Supplementary file 1 — Supplementary Material 1 [file 12885_2023_10820_MOESM1_ESM.doc]

**The Effect of Immunonutrition in Patients Undergoing Pancreaticoduodenectomy: A Systematic Review and Meta-Analysis**

Yinyin Fan^1,2^, Nianxing Li^2^, Jing Zhang^2^, Qiaomei Fu^2^, Yudong Qiu^2^, Yan Chen^1,2,*^

1. Department of Hepatobiliary Pancreatic Center, Nanjing Drum Tower Hospital Clinical College of Jiangsu University, Nanjing, 210008, China.
2. Department of Hepatobiliary Pancreatic Center, Nanjing Drum Tower Hospital, the Affiliated Hospital of Nanjing University Medical School, Nanjing, 210002, China.

*Corresponding author:

Yan Chen, Nanjing Drum Tower Hospital, the Affiliated Hospital of Nanjing University Medical School, Nanjing, China. Email: njchenyan@126.com

**Supplementary Data**


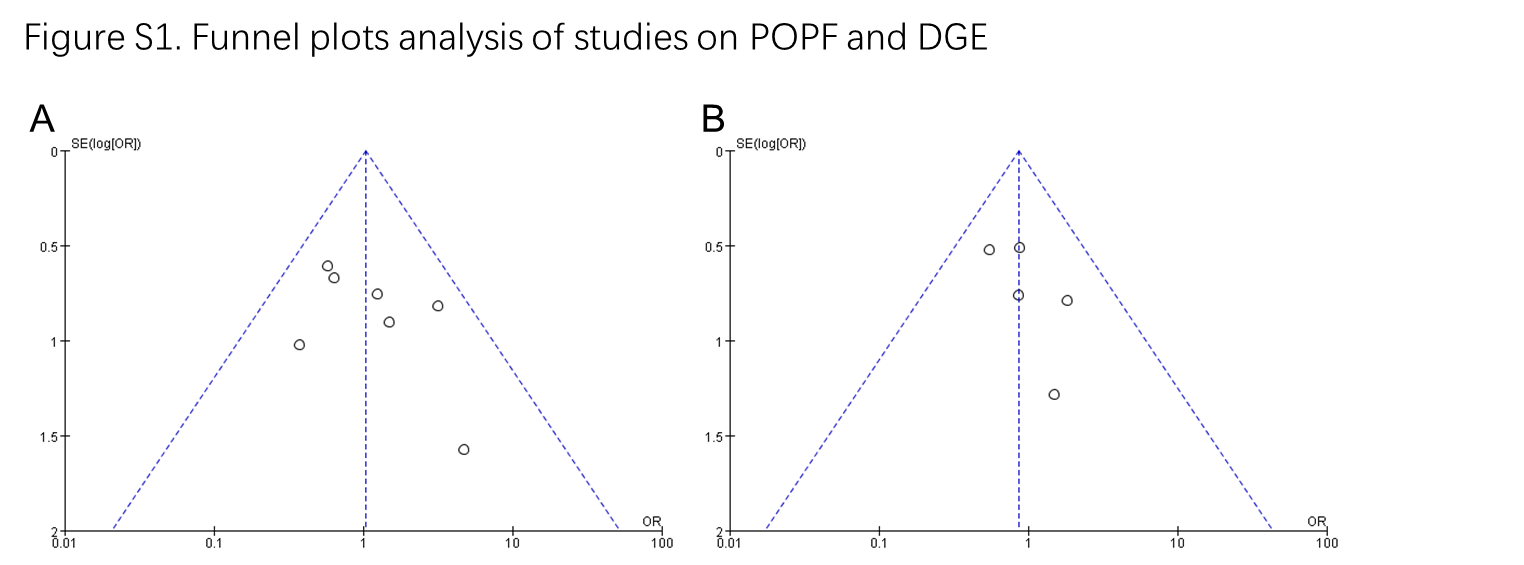


Figure S1. Funnel plots for studies investigating postoperative pancreatic fistula (POPF) and delayed gastric emptying (DGE). Panel A displays a funnel plot of all the studies related to POPF, while panel B shows the funnel plots focusing on DGE.
